# Married women’s autonomy and post-delivery modern contraceptive use in the Democratic Republic of Congo

**DOI:** 10.1186/s12905-018-0540-1

**Published:** 2018-03-12

**Authors:** Yuji Sano, Roger Antabe, Kilian Nasung Atuoye, Joseph A. Braimah, Sylvester Z. Galaa, Isaac Luginaah

**Affiliations:** 1 Department of Sociology, Western University, London, ON Canada; 2 Department of Geography, Western University, London, ON Canada; 3 Department of Social, Political and Historical Studies, Faculty of Integrated Development Studies, University for Development Studies, Tamale, Ghana

**Keywords:** Modern contraceptive, Women’s autonomy, Maternal and child health, Demographic and health survey, The Democratic Republic of Congo

## Abstract

**Background:**

Although use of modern contraception is considered beneficial in lowering maternal and child mortality rates, the prevalence of contraceptive use remains low in the Democratic Republic of Congo. This study examined modern contraceptive use and its linkage to women’s autonomy.

**Methods:**

Data were drawn from the 2013–2014 Democratic Republic of Congo Demographic and Health Survey. We selected unsterilized and non-pregnant married women who have given birth in the last three years (*N* = 6680). Logistic regression models were fitted to explore the relationship between women’s autonomy and modern contraceptive use.

**Results:**

The study found that only 7.1% of married women who had delivered within three years used modern contraceptive methods. After controlling for socioeconomic and demographic factors, the association between women’s autonomy and modern contraceptive use remained positively significant (OR = 1.16; 95% CI = 1.05, 1.29).

**Conclusion:**

The findings from this study indicate that it is not enough to provide women with educational and employment opportunities to increase the uptake of modern contraception, but also to enhance women’s assertiveness to make their own decisions regardless of their partners’ preferences within household settings. It is critical for government and other stakeholders to roll out programs aimed at reducing gender inequality and improving women’s autonomy in decision-making about reproductive health.

## Background

As unintended pregnancy and short inter-pregnancy spacing are leading causes of maternal and child death in sub-Saharan Africa, use of modern contraception after delivery is considered a critical part of interventional efforts [1, 2]. In the Democratic Republic of Congo (DRC), for example, the National Strategic Plan for Family Planning aims to increase access to modern contraceptive use from 6.3% in 2003 to 19% by 2020 [3]. Despite this effort, it is estimated in 2013–14 that only 8% of women were using modern contraception [4]. Other studies estimate modern contraceptive use to be as low as 5.7% among women aged 15–49 years [5]. The low rate of modern contraceptive use is alarming given that the overall fertility rate of the country is 6.6, with a higher regional rate of 7.7 in the Kasai-Occidental province [4]. Moreover, the 2013–14 DHS report indicated that 27% of adolescents aged 15–19 have started childbearing, and population is estimated to double every 23 years given the current trend [6]. In response, previous studies have identified important factors associated with use of modern contraception including lack of formal education [7], financial barriers [8], husband’s desire for larger family [9], rural residence [10], and province of residence [5].

In addition to these factors, women’s household decision-making autonomy (herein, women’s autonomy) is also considered important in improving women’s assertiveness to negotiate reproductive and sexual health choices with their partners [11]. Autonomy is the capacity to independently make decisions relating to one’s own life and that of other dependents with unhindered access to adequate information [12, 13]. While women’s empowerment and autonomy may both address gender inequality, these two concepts are slightly different. Although women’s empowerment is often conceptualized with socioeconomic status such as education, wealth, and employment, their autonomy is considered to be an indicator of the complex nature of gender power dynamics in decision-making within the household [14]. Women’s autonomy is important in achieving better health-related outcomes such as increased antenatal visits, reduction in overall fertility, and higher child survival rates [15–17]. Research has also found that use of modern contraception is influenced by women’s autonomy in sub-Saharan Africa including Ethiopia, Uganda, and Ghana [18–20].

Although these studies are important, the linkage between women’s autonomy and use of modern contraception is underexplored in the DRC. The country has been devastated by years of civil war and has one of the worse maternal and child mortality rates in the world [21]. The intersection of political instability and strong patriarchy could serve as a double penalty for women’s autonomy and low involvement in household decision-making. For instance, the National Reproductive Health Program has indicated that married women in the DRC are subjected to the mandates of their husbands and often do not have equal say in household decision-making including reproductive health choices [3]. In contributing to women’s reproductive health in the DRC, it is important to understand the role of women’s autonomy, which goes beyond women’s socioeconomic positioning within households. To this end, we aim to examine the impact of women’s autonomy on post-delivery contraceptive use among married women in the DRC.

## Methods

### Data and sampling

Data for this study were drawn from the 2013–14 Democratic Republic of Congo Demographic and Health Survey (CDHS). The CDHS is a nationally representative dataset of women aged 15 to 49, implemented by Ministry of Monitoring, Planning and Implementation of the Modern Revolution, in collaboration with the Ministry of Public Health. The CDHS utilized a multi-staged sampling framework in which systematic sampling with probability to size was applied to identify Enumeration Areas from which households were chosen. A total of 19,097 women were initially identified at the time of fieldwork, and 18,827 women were successfully interviewed, with a response rate of 99% [4]. For this study, we limited our sample to 6680 unsterilized and non-pregnant married women who have given birth within the last three years. This is consistent with the WHO’s [22] recommendation that women should wait at least three years to have another pregnancy after the most recent delivery.

### Dependent variable

Women were asked whether they were using any modern contraceptive method at the time of data collection, and we adopted this question as the dependent variable (0 = no; 1 = yes). The dependent variable was informed by the WHO reproductive health indicators guideline [23], suggesting that modern contraceptive methods include female and male sterilization, copper-releasing intrauterine device, hormone-releasing intrauterine device, hormonal methods (pill, patch and ring, injectable, implant), condom, and vaginal barrier methods (diaphragm, cervical cap and spermicidal agents). As shown in Tables 1, 7.1% of married women who have given birth in the last three years used modern methods.

**Table 1:** Univariate analysis of selected dependent and independent variables

	Percentage
Use of modern contraception	
No	92.9
Yes	7.1
Level of education	
No education	21.0
Primary education	45.1
Secondary education	32.4
Higher education	1.5
Wealth quintiles	
Poorest	26.3
Poorer	23.5
Middle	20.9
Richer	16.6
Richest	12.7
Currently working	
No	23.6
Yes	76.4
Age of respondents	28.0^a^ (range: 15.0 to 49.0)
Residence of province	
Kinshasa	5.5
Bandundu	13.5
Bas-Congo	4.4
Equateur	15.9
Kasai-Occidental	9.5
Kasai-Oriental	12.4
Katanga	12.7
Maniema	4.8
Nord-Kivu	5.3
Orientale	10.5
Sud-Kivu	5.5
Urban-rural residence	
Urban	29.4
Rural	70.6
Religious affiliations	
Christian	95.5
Non-Christian	4.5
Number of children	
Less than three	29.7
Three to five	40.5
More than five	29.8

^a^For age of respondents, mean and range were reported

### Independent variable

The independent variable for this study was “women’s autonomy,” which is a summative scale created with Principal Component Analysis (PCA). Specifically, PCA was used to construct a single latent scale with four questions that asked women about who usually has the final say in household settings on the following decisions: 1) obtaining their own health care, 2) making large purchases for daily needs, 3) visits to family and relatives, and 4) household expenditures (0 = husband alone; 1 = respondent and husband together; 2 = respondent alone). Cronbach’s alpha for this scale was statistically robust (α = 0.75). All indicators were loaded on a single latent scale construct with factor loadings ranged from 0.70 to 0.79. Higher values on this scale indicate that women have higher autonomy, while lower values indicate lower autonomy. This approach has been employed in previous studies (see [24–26]).

### Control variables

In this study, two blocks of control variables were introduced, namely socioeconomic and demographic variables. Specifically, socioeconomic variables include level of education (0 = no education; 1 = primary education; 2 = secondary education; 3 = higher education), household wealth quintiles (0 = poorest; 1 = poorer; 2 = middle; 3 = richer; 4 = richest), and employment status (0 = not employed; 1 = employed). Household wealth quintiles were constructed from a composite index based on the household ownership of consumer items, such as drinking water, car, and toilet facilities, among others. Moreover, we controlled for four demographic variables such as age of respondents (measured in completed years), province of residence (0 = Kinshasa; 1 = Bandundu; 2 = Bas-Congo; 3 = Equateur; 4 = Kasai-Occidental; 5 = Kasai-Oriental; 6 = Katanga; 7 = Maniema; 8 = Nord-Kivu; 9 = Orientale; 10 = Sud-Kivu), urban-rural residence (0 = urban; 1 = rural), religious affiliations (0 = Christian; 1 = non-Christian), and number of children (0 = less than three; 1 = three to five; 2 = more than five).

### Statistical analysis

In addition to descriptive analysis of selected dependent and independent variables, logistic regression models were sequentially built. Specifically, we examined the bivariate association between women’s autonomy and use of modern contraception in Model 1, while Models 2 and 3 controlled for socioeconomic and demographic factors, respectively. This may be expressed as the following:

$$ \ln \left(\frac{p}{1-p}\right)=\alpha +{\beta}_1{x}_1+{\beta}_2{x}_2+\dots +{\beta}_k{x}_k $$

where *p* is the probability that postpartum married women were using modern contraception, *α* is the constant, *k* is the total number of independent and control variables, *β*_1_, *β*_2_, …, *β*_*k*_ are regression coefficients, and *x*_1_, *x*_2_, …, *x*_*k*_are independent and control variables [27]. Moreover, there is usually some degree of dependence among subjects in surveys with the multistage sampling framework including the CDHS. As this could potentially violate the assumption of independence among subjects, a ‘cluster’ variable (ID numbers) was introduced in models, which adjusted standard errors. For meaningful interpretations, results were reported with odds ratios. Odds ratios larger than one indicate that women were more likely to use modern contraception, while odds ratios smaller than one indicate lower odds of doing so.

## Results

### Univariate analysis

Findings from univariate analysis are shown in Table 1. More than one third of women (33.8%) had secondary or higher education. Although 76.4% of the respondents were employed, about half of them were from the poorest (26.3%) and poorer households (23.5%). Moreover, the largest proportion of respondents lived in Equateur (15.9%), followed by Bandundu (13.6%), Katanga (12.7%), and Kasai-Oriental (12.4%). Majority of women lived in rural areas (70.6%), were Christians (95.5%), and had at least three children (70.3%).

### A closer look at women’s autonomy

We explored percentage distributions of four questions related to women’s decision-making autonomy in Table 2. While less than 9% of women indicated that they had the ability to independently make decisions about their own health care (8.8%) and household expenditures (8.6%), approximately 15% reported that they can independently make large purchases for daily needs (14.6%) and visit to their families and relatives (16.2%).

**Table 2:** Descriptive analysis of women’s autonomy

	Percent		
	Only husband	Respondent and husband	Only respondent
Own health care	55.4	35.8	8.8
Making large purchases	42.8	42.7	14.5
Visits to family/relatives	48.9	34.9	16.2
Household expenditure	45.7	45.7	8.6
Women’s autonomy	0.1^a^ (range: −1.2 to 2.7)		

^a^For women’s autonomy, median and range were reported

### Regression analysis

Table 3 shows results from the multivariate analysis. At the bivariate level, we found that the relationship between women’s autonomy and use of modern contraception is statistically significant (OR = 1.28; 95% CI = 1.18, 1.40), indicating that married women with higher levels of autonomy were more likely to use modern contraception than their counterparts with lower levels of autonomy. Although its significant impact remained robust after controlling for socioeconomic variables in Model 2, it was partially mediated in Model 3 when demographic variables were controlled (OR = 1.16; 95% CI = 1.05, 1.29). A further analysis (result not shown here) indicates that it was urban-rural residence that attenuated the impact of women’s autonomy on modern contraceptive use.

**Table 3:** Logistic regression models of “use of modern contraception” among married women in the DRC

	Model 1	Model 2	Model 3
	OR [95% CI]	OR [95% CI]	OR [95% CI]
Women’s autonomy	1.28 [1.18,1.40]***	1.19 [1.08,1.31]***	1.16 [1.05,1.29]**
Level of education			
No education		1.00	1.00
Primary education		1.31 [0.91,1.88]	1.18 [0.82,1.70]
Secondary education		2.82 [1.95,4.07]***	2.41 [1.65,3.51]***
Higher education		3.29 [1.75,6.17]***	2.90 [1.50,5.58]**
Wealth quintiles			
Poorest		1.00	1.00
Poorer		1.59 [1.09,2.32]*	1.48 [1.01,2.17]*
Middle		1.78 [1.23,2.60]**	1.46 [0.99,2.14]
Richer		3.06 [2.13,4.40]***	1.86 [1.26,2.75]**
Richest		4.94 [3.39,7.21]***	2.18 [1.36,3.49]**
Currently working			
No		1.00	1.00
Yes		1.00 [0.80,1.24]	1.00 [0.79,1.26]
Age of respondents			0.97 [0.95,0.99]*
Residence of province			
Kinshasa			1.00
Bandundu			1.05 [0.68,1.60]
Bas-Congo			2.82 [1.81,4.39]***
Equateur			0.42 [0.25,0.70]***
Kasai-Occidental			0.66 [0.40,1.08]
Kasai-Oriental			0.51 [0.32,0.80]**
Katanga			0.46 [0.30,0.71]***
Maniema			0.61 [0.33,1.11]
Nord-Kivu			1.26 [0.80,2.00]
Orientale			0.75 [0.47,1.19]
Sud-Kivu			0.77 [0.44,1.35]
Urban-rural residence			
Urban			1.00
Rural			0.44 [0.33,0.58]***
Religious affiliations			
Christian			1.00
Non-Christian			1.30 [0.82,2.06]
Number of children			
Less than three			1.00
Three to five			1.03 [0.79,1.35]
More than five			1.30 [0.87,1.94]
Wald chi-square	33.19 (1)***	332.07 (9)***	402.35 (24)***
Pseudo r-square	0.01	0.10	0.13
Log pseudo-likelihood	− 1701.6	− 1550.5	− 1490.8

A crude odds ratio for Model 1; Adjusted odds ratios in Models 2 and 3

*OR* for odds ratios, *95% CI* for 95% confidence internals

* *p* < 0.05, ** *p* < 0.01, *** *p* < 0.001

In addition to women’s autonomy, there were other theoretically relevant variables significantly associated with use of modern contraception. For example, women with secondary (OR = 2.41; 95% CI = 1.65, 3.51) and higher education (OR = 2.90; 95% CI = 1.50, 5.58) were more likely to use modern contraception than those with no education. Similarly, women from the richest (OR = 2.18; 95% CI = 1.36, 3.49), richer (OR = 1.86; 95% CI = 1.26, 2.75), and poorer households (OR = 2.18; 95% CI = 1.36, 3.49) were more likely to use modern contraception than those from the poorest households. Also, compared to those in Kinshasa, women in Equateur (OR = 0.42; 95% CI = 0.25, 0.70), Kasai-Oriental (OR = 0.51; 95% CI = 0.32, 0.80), and Katanga (OR = 0.46; 95% CI = 0.30, 0.71) were less likely to use modern contraception. Women in rural areas were also less likely to use modern contraception than their urban counterparts (OR = 0.44; 95% CI = 0.33, 0.58).

## Discussion

Within the unique context of prolonged conflict and strong patriarchy where husbands have persistent desire and preference for larger family size [9], we found that modern contraceptive use in the DRC was influenced by women’s autonomy. Other studies found this linkage in sub-Saharan Africa [18–20], suggesting that some women may face barriers to use modern contraception due to limited decision-making autonomy, which probably reflects unequal power dynamics between women and men within households. In the particular context of the DRC, we argue that years of civil war could have watered-down women’s autonomy and limited their ability to negotiate for modern contraceptive use [28]. This finding points to the importance of looking beyond women’s socioeconomic empowerment such as education, wealth, and employment to understand use of modern contraception in the DRC.

In addition to women’s autonomy, it is important to discuss other factors that were significantly associated with use of modern contraception. For example, attainment of higher levels of education was related to use of modern contraception. This lends support to Shapiro and Tambashe [7], suggesting that pronatalist culture in the DRC may limit women’s use of modern contraception, and less educated women may be particularly vulnerable to low use of modern contraception. The result that wealthier women were more likely to use modern contraception than poorer women is consistent with results from previous research [8]. Given that almost three-quarters (71.3%) of the general population live below the poverty line and a further 45.9% are classified as living in severe poverty in the DRC [6], it may not be surprising that married women who are further economically disadvantaged in this patriarchal society are unable to afford modern contraception [5].

Related to demographic factors, we found that province of residence strongly influenced women’s use of modern contraception. Despite the fact that the DRC has been ravaged by war and there still persists pockets of instability, the western provinces are the least affected and have a relatively better socioeconomic infrastructure and higher access to basic healthcare than the rest of the country [28, 29]. In addition, there are a number of non-governmental organizations that provide access to modern contraception in the western part of the country [30]. These may explain why women in the war-torn eastern provinces such as Equateur, Kasai-Oriental, and Katanga were less likely to use modern contraception.

Similarly, women in rural areas were less likely to use modern contraception than their urban counterparts, reflecting wide rural-urban disparities in access to health care and level of awareness on modern contraceptive use. Additionally, patriarchy and other cultural values such as the strong desire for high fecundity dominant in rural settings may serve as barriers to the use of modern contraception [31]. This is consistent with our findings that the relationship between women’s autonomy and modern contraceptive use was partially attenuated when urban–rural residence was controlled. Studies elsewhere indicate that women in urban areas may be more likely to use modern contraception due to close proximity to health facilities and related services [8, 10].

Although these findings may be useful for policymakers, there are noteworthy limitations. First, due to the cross-sectional nature of the CDHS, our interpretations of results are limited to statistical associations. Moreover, due to the sensitive nature of contraceptive use, the reliability of self-reported surveys such as the CDHS may be questionable with a possible underreporting issue. For future research, use of qualitative and longitudinal data may better explain the relationship between women’s autonomy and use of modern contraceptive methods. Despite these limitations, this study is one of few attempts to explore the effect of women’s autonomy on use of modern contraception in three years of post-delivery in the DRC.

## Conclusions

Understanding women’s autonomy as an important predictor of post-delivery modern contraceptive use is critical for informed policy on improved maternal and child health in the DRC. As suggested by our study findings, educational and employment opportunities alone may not be enough to increase the uptake of modern contraception. It is also important to enhance women’s assertiveness to make their own decisions regardless of their partners’ preferences within household settings. Therefore, it is critical for government and other stakeholders to roll out programs aimed at reducing gender inequality and improving women’s assertiveness in decision-making within households. In addition, it is suggested that interventional programs need to focus on providing universal access and awareness to modern contraceptive services, especially among women with low level of education, and those from poorer households in rural and conflict-affected areas in the DRC [5].

## Abbreviations

CDHS: Democratic Republic of Congo Demographic and Health Survey
DRC: Democratic Republic of Congo
PCA: Principal Component Analysis
WHO: World Health Organization
